# Antiresorptive Use and Risk of Hip Fracture in Older Women with Osteoporosis: Findings from a Real-World National Database of 21,332 Women

**DOI:** 10.3390/jcm15166224

**Published:** 2026-08-11

**Authors:** Bernardo A. Cedeno-Veloz, Juan Erviti, Marta Gutiérrez-Valencia, Leire Leache, Luis Carlos Saiz, Alba M. Rodríguez García, Nicolás Martínez-Velilla

**Affiliations:** 1 Geriatric Department, Hospital Universitario de Navarra (HUN), c. Irunlarrea, 31008 Pamplona, Spain; 2 Navarrabiomed, Navarra Institute for Health Research (IdiSNA), 31008 Pamplona, Spainmarta.gutierrez.valencia@navarra.es (M.G.-V.); alba.rodriguez.garcia@navarra.es (A.M.R.G.); 3 Department of Health Science, Public University of Navarre, av. Cataluña s/n, 31006 Pamplona, Spain; 4 CIBER of Frailty and Healthy Aging (CIBERFES), Instituto de Salud Carlos III, av. Monforte de Lemos, 5, 28029 Madrid, Spain; 5 Unit of Innovation and Organization, Navarre Health Service, c. de Tudela 20, 31003 Pamplona, Spain; 6 Pharmacy and Services Sub-Directorate, Navarre Health Service, c. de Tudela 20, 31003 Pamplona, Spain

**Keywords:** older women, osteoporosis, antiresorptive drugs, hip fracture, real-world evidence

## Abstract

**Background**: Evidence on the real-world effectiveness of antiresorptive therapy for hip fracture prevention in very old women remains limited. We aimed to assess the association between antiresorptive use and incident hip fracture among age ≥ 75 older women with osteoporosis. **Methods**: We conducted a population-based nested case–control study using the Base de Datos para la Investigación Farmacoepidemiológica en Ámbito Público (BIFAP), a Spanish research database. Women aged 75 years or older with osteoporosis and no previous antiresorptive use at cohort entry were followed from 2010 to 2022. Incident hip fracture cases were matched to risk-set controls by age, autonomous region, and calendar year of database registration. Antiresorptive exposure was classified as current, recent, past, or never use. Conditional logistic regression was used to estimate adjusted odds ratios (aORs) and confidence intervals. **Results**: The study included 2057 incident hip fracture cases and 19,275 matched controls. Compared with never users, current antiresorptive users had lower odds of hip fracture (aOR 0.76; 0.65–0.87), whereas past users had higher odds (aOR 1.91; 1.57–2.33). Ever use was not associated with hip fracture risk compared with never use (aOR 1.01; 0.90–1.13). **Conclusions**: In this real-world nested case–control study of women aged ≥75 years with osteoporosis, current antiresorptive use was associated with lower odds of hip fracture, whereas past use was associated with higher odds. Because treatment initiation, persistence, and discontinuation are strongly influenced by baseline fracture risk, osteoporosis severity, frailty, and clinical surveillance, these findings should be interpreted as associations rather than causal treatment effects.

## 1. Introduction

Osteoporosis (OP) is an age-related syndrome that has been associated with poor outcomes, such as disability, an increased risk of falls and fractures, loss of independence, high costs to healthcare systems, and an increased risk of premature death [1]. Of the various osteoporotic fractures, hip fractures are particularly severe, given their high mortality rate, substantial economic burden, and profound personal and social consequences for those affected [2]. In Spain, for individuals aged over 60, there was an estimated prevalence of 7.20 hip fractures per 1000 inhabitants/year in 2003 [3].

Over the past 50 years, major efforts have advanced OP research, risk assessment tools, and non-invasive diagnostic methods. However, the ability of these tools to predict hip fracture risk remains limited. Despite initiatives from scientific societies, patient associations, and government strategies, challenges in effective OP management persist [4,5]. Despite these relentless efforts, managing the progression of OP, referred to as a “silent epidemic” [1] affecting the global population, remains elusive. Projections for OP are far from optimistic. By 2050, due to ongoing aging and the lack of effective management measures, hip fracture rates are projected to increase in Europe by nearly 46% [6]. Similarly discouraging projections apply to other regions worldwide. The primary challenge is to implement effective primary prevention, focusing on enhancing and maintaining bone health to prevent the loss of bone mass and alterations in microarchitecture, which increase bone fragility and fracture risk [7].

The introduction of bisphosphonates to the market demonstrated efficacy in enhancing bone density, albeit without clear evidence of reducing hip fractures [8]. They were introduced based on the theoretical assumption that increased bone density would fortify bone structure and thus diminish fracture risk. Antiresorptives are frequently the first-line treatment for secondary prevention of OP in older adults [4,5,9].

However, in most pivotal trials comparing the effects of these drugs to no treatment, hip fractures were considered secondary endpoints, with results showing no definitive advantage in reducing hip fracture risk [10]. Several meta-analyses have reported statistically significant benefits of these drugs over placebo, yet the clinical significance of these findings remains debatable [11], and methodological biases are evident in these reviews [12]. Furthermore, some recent analyses reveal a high number needed to treat with prolonged exposure time [13]. Prolonged use of antiresorptives has been associated with adverse effects on bone structure, including osteonecrosis of the jaw, atypical fractures, and bone pain. This has led to several safety advisories from regulatory agencies [14,15].

Given the intricacies and economic costs associated with conducting new clinical trials, cohort studies emerge as an alternative to investigate the effectiveness of these drugs in fracture prevention [16]. These cohort studies have been periodically published, yielding both favorable [17,18,19] and unfavorable [20,21,22] results regarding their use for post-hip fracture prevention, leaving a critical question unanswered. Furthermore, the evidence is sparse in older populations, who are at the highest risk of hip fractures. We hypothesized that the association between antiresorptive use and hip fracture would vary according to the timing and duration of exposure, particularly in very old women treated in routine clinical practice.

Thus, our objective was to estimate the real-world association between patterns of antiresorptive use and incident hip fracture among women aged ≥75 years with osteoporosis. Specifically, we aimed to evaluate both the overall effect of medication exposure (compared to never users) and the risks associated with treatment discontinuation (by comparing current use against recent and past exposure).

## 2. Materials and Methods

### 2.1. Study Design and Data Source

A population-based case–control study nested in a cohort was carried out. It was performed in the Spanish primary care population-based research database BIFAP (Base de Datos para la Investigación Farmacoepidemiológica en Ámbito Público, Database for Pharmacoepidemiologic Research in Public Care). BIFAP is a non-profit program of the Spanish Agency of Medicines and Medical Devices, which collaborated with nine autonomous regions of Spain when data were extracted (www.bifap.org) [23].

The BIFAP database contains comprehensive health information on over 24 million individuals, including demographics, clinical records, prescriptions (classified by ATC), and lifestyle data. Its data accuracy is validated through internal checks and prior studies [23]. This study, with retrospective data observation period from 1 January 2010 to 31 December 2022, adheres to the STROBE guidelines for cohort research [24].

### 2.2. Study Cohort and Follow-Up

**Figure 1:**
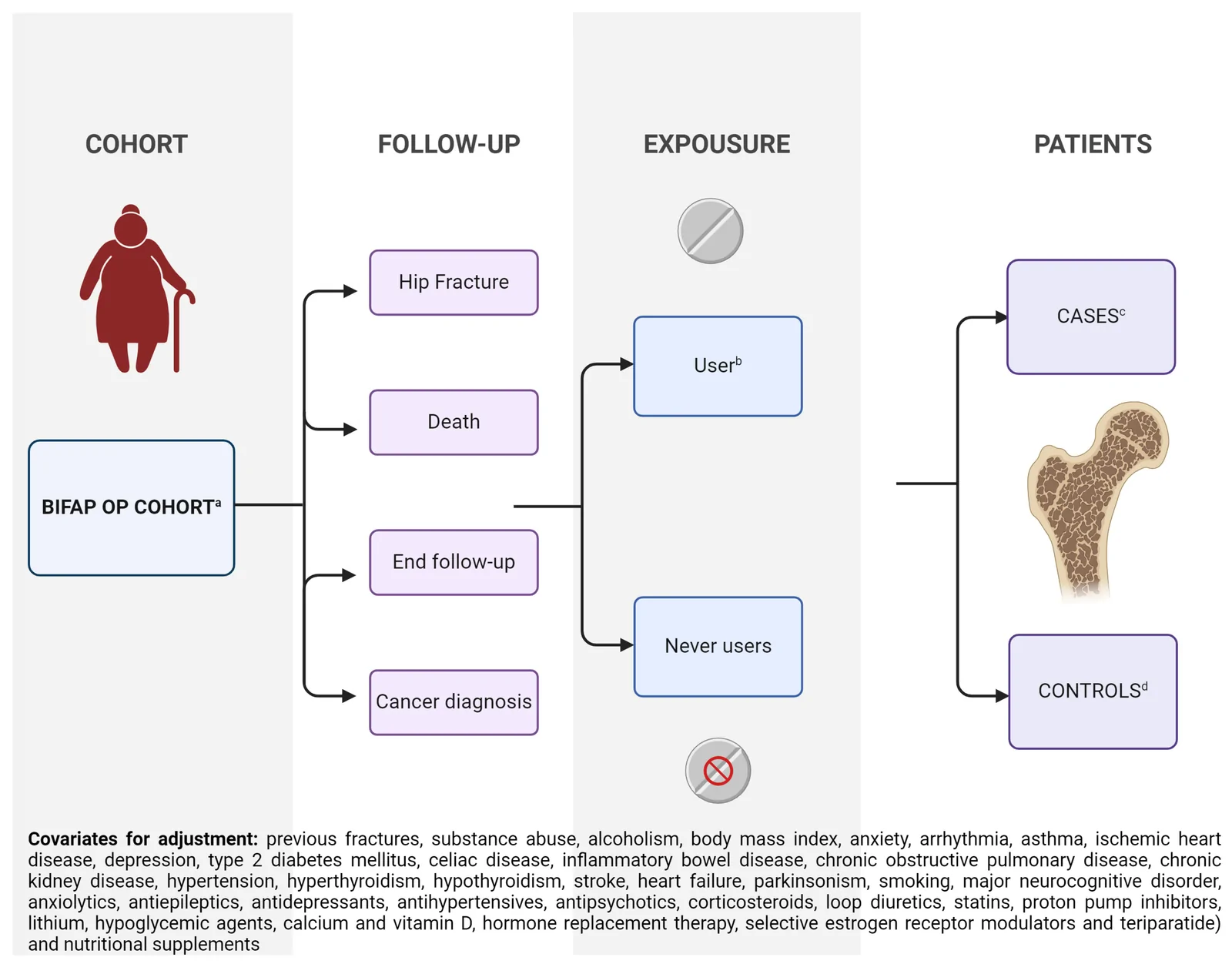
Study design and criteria. ^a^ Cohort criteria: women 75 or older with a diagnosis of osteoporosis, naive to antiresorptive use, and records in BIFAP between 1 January 2010 and 31 December 2021 with at least one year of follow-up in BIFAP prior to their entry into the cohort. Excluded patients had a history of cancer (excluding basal cell skin cancer), Paget’s disease, rheumatoid arthritis, polymyalgia rheumatica, or ankylosing spondylitis, a history of hip fracture from high-impact trauma, and patients on oral corticosteroid treatment for more than 3 months. ^b^ Cases: hip fracture event during the study period and one year of follow-up before it. ^c^ Controls: no hip fracture on the index date, and at least one year of follow-up prior to the index date. Matched by sex (only women), age (years; ±1 year), autonomous community, and calendar year of registration in BIFAP. ^d^ Three different groups were considered: current users (exposed to antiresorptives on the index date or when ≤1 year had elapsed since the last prescription), recent users (1–3 years had passed since the last prescription), and past users (>3 years had passed since the last prescription).

The cohort (Figure 1) was comprised of women aged 75 or older with a diagnosis of osteoporosis (Table S1), naive to antiresorptive use. The ‘cohort entry date’ for each patient was defined as the date of their first osteoporosis diagnosis with an electronic record in BIFAP between 1 January 2010 and 31 December 2021. To be eligible, patients were required to have at least 365 days of continuous registration in the BIFAP database immediately preceding their cohort entry date to ensure adequate baseline data. Patients with a history of cancer (excluding basal cell skin cancer), Paget’s disease, rheumatoid arthritis, polymyalgia rheumatica, or ankylosing spondylitis, a history of hip fracture from high-impact trauma, and patients on oral corticosteroid treatment for more than 3 months were excluded.

Following cohort entry, all eligible subjects were followed until the earliest occurrence of one of the following endpoints: an incident hip fracture (the main study event), cancer diagnosis (excluding basal cell skin carcinoma), death from any cause, loss to follow-up, or the end of the study period (31 December 2022). To guarantee sufficient time-at-risk, we applied an additional criterion: both potential cases and their matched controls were required to have at least one year of active follow-up time strictly between their cohort entry date and their index date.

Potential cases were included as cases in those patients with a hip fracture event during the study period and, at least, had one year of follow-up before the main study event (hip fracture). The index date was considered as the day of hospitalization in case of event registration in CMBD (the Register of Specialised Care Activity) or the day of registration in medical history if the event was only recorded in primary care (Table S2).

Up to 10 controls were selected per case. These subjects did not have a hip fracture on the index date corresponding to their case and should also have at least one year of follow-up prior to the index date. Controls were matched with cases based on age (years; ±1 year), region, and calendar year of registration in BIFAP.

A validation of potential cases was carried out. After validating the medical records of a randomly selected sample of 200 potential cases, only 1 was considered not a true case (positive predictive value: 99.8%), being excluded from the study cohort together with its matching controls. Given the high validity of the hip fracture definition, no further adjudication was considered necessary.

### 2.3. Definition of Exposures

The main exposure was the prescription of antiresorptive drugs according to ATC codes of antiresorptives (Table S3): alendronate, clodronate, denosumab, etidronate, ibandronate, pamidronate, risedronate, tiludronate, or zoledronate. Patients who received these drugs at any time before the index date (with no backward date limit) were considered as “user”.

Those women who had not received treatment with antiresorptive drugs before the index date (with no backward date limit) were considered as “never users”. Prescriptions with on-demand or conditional administration were not considered.

Within the group of users, the following were considered (Figure S1A):

“Current users”: patients exposed to an antiresorptive on the index date or when ≤1 year has elapsed since the last prescription until the index date.

“Recent users”: between 1 year and 3 years have elapsed since the last prescription until the index date.

“Past users”: more than 3 years have elapsed since the last prescription until the index date.

To analyze the effect of exposure time to antiresorptives, the “continuous duration” was determined, referring to the total number of uninterrupted days the patient was taking antiresorptive treatment (allowing a gap of 90 days between the end of one prescription and the start of the next one, and coverage of 183 days for denosumab administrations and 365 days for zoledronate infusions). Similarly, the “accumulated duration” was established, understood as the summation of the total days the patient received antiresorptives, whether continuously or not, prior to the index date. Based on this, the following categories were established: ≤1 year, >1 year and <3 years, and ≥3 years (Figure S1B).

### 2.4. Other Covariates’ Definitions

In addition to the exposure variable and the event, the following information was collected, as they constitute potential confounding factors: previous fractures (including hip fractures occurring strictly prior to the cohort entry date), substance abuse, alcoholism, body mass index, history of anxiety, arrhythmia, asthma, ischemic heart disease, depression, type 2 diabetes mellitus, celiac disease, inflammatory bowel disease, chronic obstructive pulmonary disease, chronic kidney disease, hypertension, hyperthyroidism, hypothyroidism, stroke, heart failure, parkinsonism, smoking, and major neurocognitive disorder. Likewise, the administration of drugs related to hip fracture was analyzed (Table S4). The presence of these factors was analyzed up to the index date.

### 2.5. Statistical Methods and Outcome Measure

The baseline characteristics of cases and controls were analyzed. Categorical variables were described through frequency distribution. In the case of quantitative variables, normality of these variables was analyzed. Measures of central tendency and dispersion were described as mean and standard deviation in the case of normal quantitative variables.

In the main analysis, conditional logistic regression was utilized to estimate the adjusted OR and 95% CI for the association between antiresorptive use and hip fractures. To provide a comprehensive evaluation, our analysis was structured around two primary reference groups: First, to evaluate the overall effect of medication exposure, the risk of hip fracture was compared across current, recent, and past users using ‘never users’ as the standard reference group. Ever users (any past or present exposure) were also compared against never users. Second, to evaluate the risks associated with treatment discontinuation, recent and past users were compared using ‘current users’ as the reference group. Likewise, continuous and accumulated duration of treatment were evaluated to identify exposure trends, again utilizing ‘never users’ as the reference group. The established significance level was *p* = 0.05. An analysis of unadjusted and adjusted results by confounding mentioned factors was performed. Additionally, the main outcome was analyzed by subgroup of antiresorptives (bisphosphonates, denosumab, and switchers). A subgroup analysis was also performed based on the history of previous fractures (including hip fracture) and the age range ≥ 85 years. Likewise, a stratified analysis was based on the duration from the diagnosis of osteoporosis until the occurrence of the first case of hip fracture. This was performed for diagnosis < 2 years, 2–5 years, and >5 years. For stratified analyses in which matched sets were broken, unconditional logistic regression was used, adjusted for the original matching variables: age at index date, autonomous region, calendar year of BIFAP registration, and length of valid BIFAP record before the index date.

## 3. Results

### 3.1. Description of the Study Cohort

**Figure 2:**
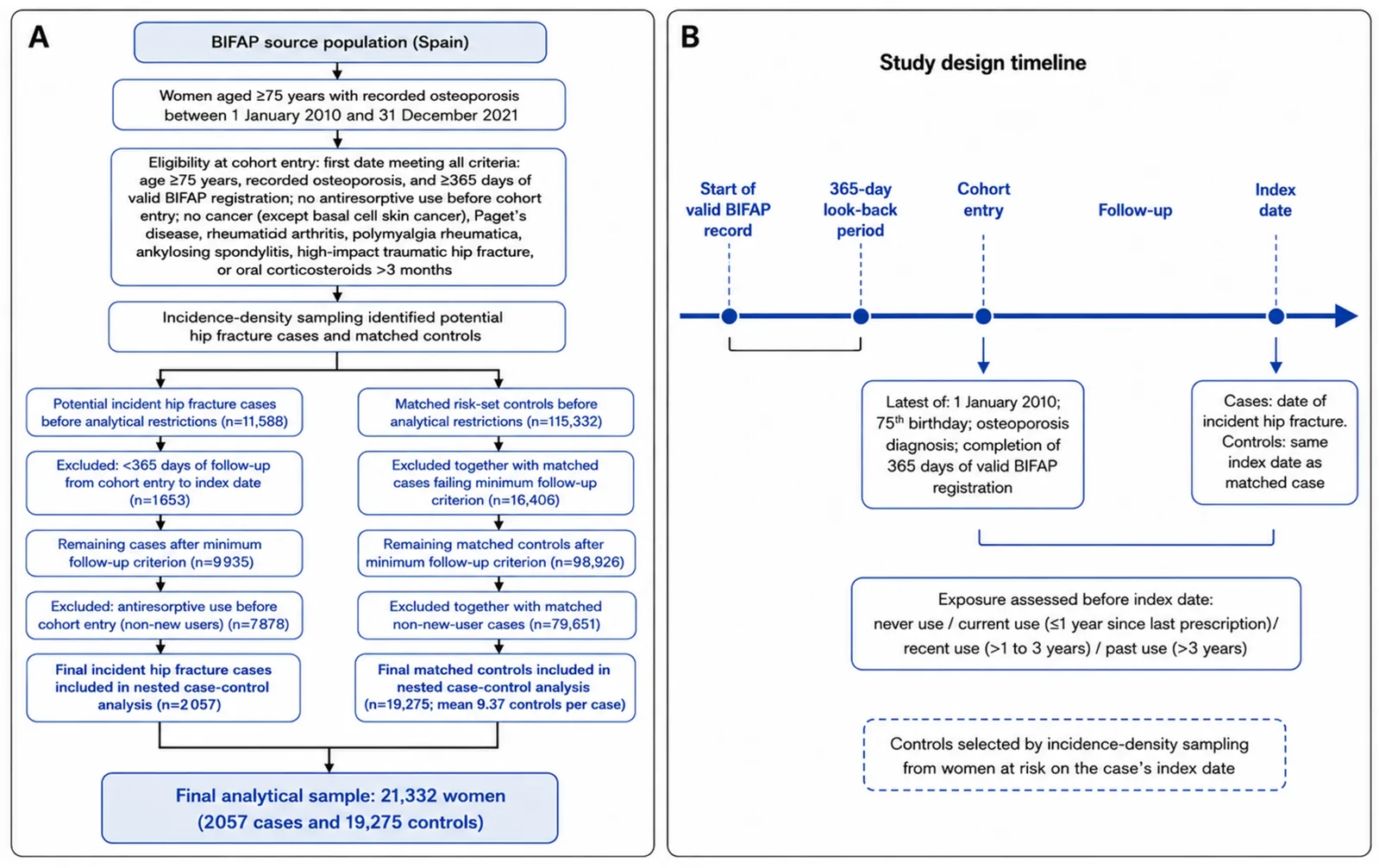
Study flow and design of the nested case–control study. (**A**) The selection of incident hip fracture cases and matched risk-set controls from the BIFAP osteoporosis cohort. (**B**) The study timeline, including the 365-day look-back period, cohort entry, follow-up, index date assignment, and classification of antiresorptive exposure before the index date. Controls were selected by incidence-density sampling among women at risk on the matched case’s index date. BIFAP: Base de Datos para la Investigación Farmacoepidemiológica en Ámbito Público.

The initial cohort had 11,588 cases and 115,332 controls. A total of 1653 cases had a follow-up period of less than 365 days before the main study event so, after the removals, the cohort had 9935 cases and 98,926 controls. After removing non-naive patients and their matched controls, 2057 cases and 19,275 controls remained (mean 9.37 controls per case). During follow-up, 2057 incident hip fracture cases were identified and validated, and 19,275 controls were selected using incidence-density sampling (Figure 2). At the index date, 301 cases and 4039 controls were classified as current antiresorptive users, 150 cases and 857 controls as recent users, 216 cases and 618 controls as past users, and 1390 cases and 13,761 controls as never users. Characteristics of cases and controls at the index date are described in Table 1. The mean age was similar between groups (87.5 vs. 87.2 years; *p* = 0.007), though cases had a significantly longer follow-up (86.6 vs. 52.8 months; *p* < 0.001). Comorbidities were more prevalent among cases (74.7% vs. 70.7%; *p* < 0.001), with statistically significant differences for atrial fibrillation, dementia, depression, stroke, and tobacco use.

Regarding medication use, cases had a higher consumption of benzodiazepine-derived anxiolytics (64.2% vs. 51.2%), antidepressants (51.4% vs. 41.6%), antipsychotics (24.9% vs. 20.7%), systemic corticosteroids (21.0% vs. 15.6%), statins (44.2% vs. 37.9%), and proton pump inhibitors (80.8% vs. 72.1%; all *p* < 0.001). They also showed higher exposure to antiresorptive treatments, such as bisphosphonates (26.1% vs. 18.8%), particularly alendronate, ibandronate, and risedronate (all *p* < 0.001), while exposure to denosumab was higher in controls (10.5% vs. 7.7%; *p* < 0.001).

Fracture prevalence was generally low, though significantly higher in cases for humeral fractures (5.8% vs. 4.4%; *p* = 0.004). No significant differences were observed in vertebral, hand, or foot fractures.

### 3.2. Primary and Secondary Analyses

**Figure 3:**
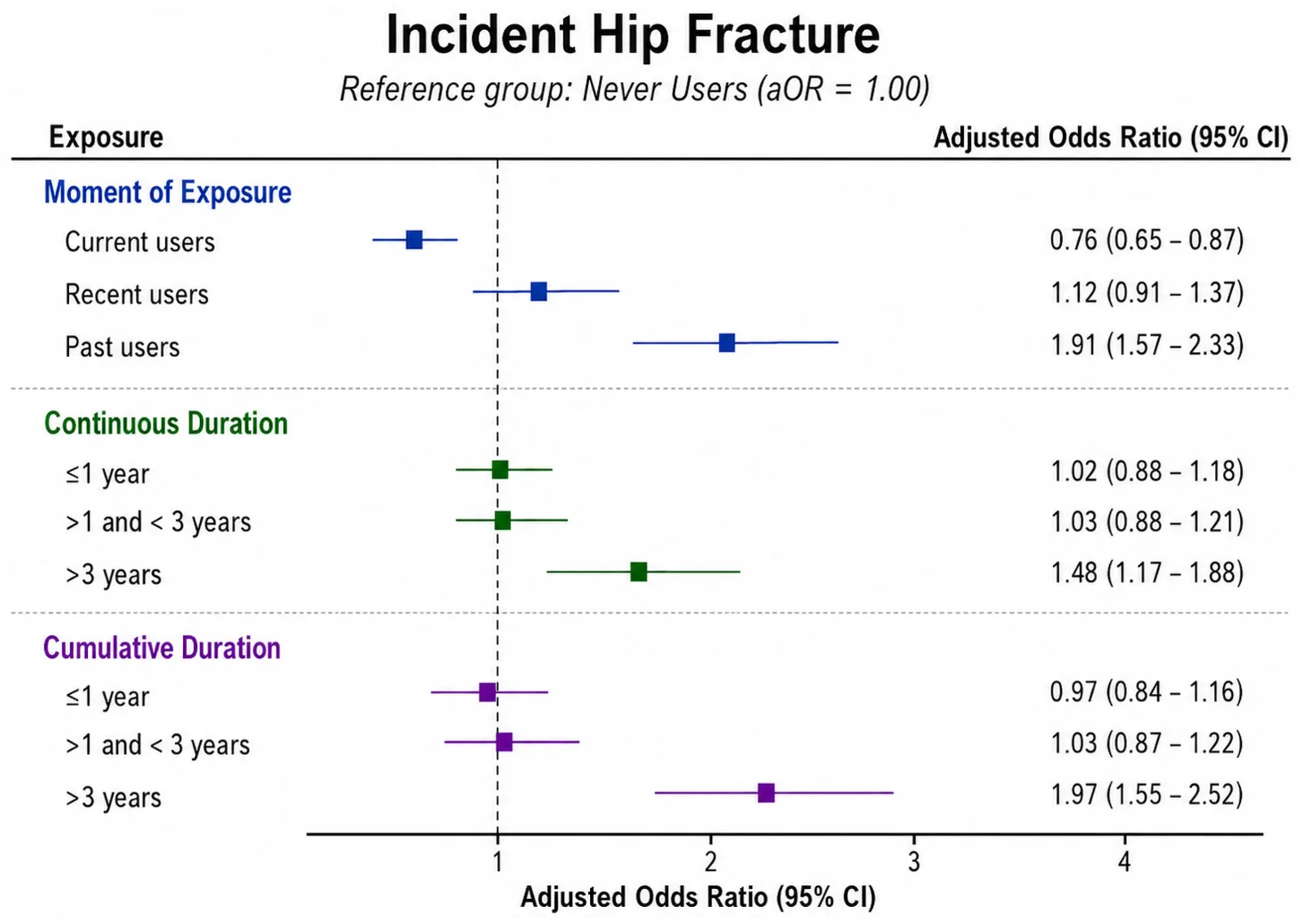
Adjusted odds ratios for incident hip fracture according to the timing and duration of antiresorptive exposure. Forest plot illustrating the adjusted odds ratios (aORs) and 95% confidence intervals for incident hip fracture according to the moment of exposure, continuous duration, and cumulative duration of antiresorptive therapy. All comparisons utilize ‘never users’ as the baseline reference group (aOR = 1.00). Current users are defined as having ≤1 year since their last prescription, recent users as >1 to 3 years, and past users as >3 years.

Regarding the main analysis (Table 2, Figure 3), when current antiresorptive users (CUs) were compared to a combined group of never users (NUs) and past users (PUs), this combined non-active exposure group showed an increased risk of hip fracture with an aOR of 1.43 (95% CI: 1.24–1.65; *p* < 0.001). Similarly, when current users were compared to a pooled group of all other categories (never, recent, and past users combined), these non-current users were also associated with an increased risk of hip fracture with an aOR of 1.35 (95% CI: 1.17–1.55; *p* < 0.001). However, when patients who had been exposed to antiresorptive drugs at any time were analyzed against those who had never been exposed, this difference disappeared (aOR 1.01; 95% CI: 0.90–1.13). According to the timing of exposure to antiresorptives, CUs showed a decreased risk of hip fracture when compared to recent, PUs, or NUs. Compared to NUs, CUs were the only group that exhibited lower odds of hip fracture (aOR 0.76; 95% CI: 0.65–0.87; *p* < 0.001), while PUs showed an increased risk (aOR 1.91; 95% CI: 1.57–2.33). Regarding the duration of exposure to antiresorptives, comparing with NUs, none of the groups showed a reduced risk of hip fracture, and a >3 year continuous or cumulative exposure was even associated with an increased risk. Concerning the interval between osteoporosis diagnosis and index date, non-current users with less than 2 years from diagnosis (aOR 1.48; 95% CI: 1.10–2.00; *p* < 0.001) and 2–5 years (aOR 1.34; 95% CI: 1.06–1.69; *p* < 0.001) had an increased risk of fracture compared to current users, with no differences observed for those with a >5 year interval from diagnosis.

### 3.3. Subgroup Analysis

Regarding secondary prevention (Table 3) and very older patients (aged ≥85 years; Table S5), the results are comparable to those of the overall population analysis, although some results are no longer statistically significant due to the reduced sample size. Furthermore, in the age ≥ 85 years subgroup, in the comparison between ever users and NUs, an increased risk was observed in the former.

When subgroups of antiresorptives were analyzed, they generally showed results with the same trends as the overall results (Tables S6 and S7). The main difference between the different antiresorptives was that in the comparison of bisphosphonate users versus never users there was an increased odds with treatment, whereas with denosumab there was a decreased odds in this comparison. Also, in the case of denosumab, those of continuous or cumulative duration of one year or less had a lower risk than never users. In switchers (Table S8), the smallest group, no differences in odds in the main analysis were seen, and the other results were not statistically significant either.

## 4. Discussion

In this large cohort of older women with osteoporosis, the comparison of receiving antiresorptive treatment showed the following: (1) current antiresorptive treatment was associated with lower odds of hip fracture compared to past and never exposed groups, (2) the comparison of those who received treatment at some point versus those never exposed showed no difference between the groups, (3) the lower odds of hip fracture observed in current users disappeared once the treatment had been discontinued for more than one year, (4) more than three years of continuous or cumulative duration was associated with higher odds of hip fractures compared to never use, and (5) hip fracture odds did not differ between groups after five years of osteoporosis diagnosis. Subgroup analyses in patients with previous fractures and in those over 85 years of age showed similar results to the overall population, as did the separate analyses of bisphosphonates and denosumab. Nonetheless, some results showed a loss of statistical power due to the small number of cases in each subgroup.

The findings indicate that antiresorptives may have an initial benefit on fracture prevention [25] but it is not maintained over the long term, irrespective of whether treatment is continued or discontinued [26]. An elevated odds of hip fracture has been observed in both treatment durations exceeding three years and in past users of more than three years, in comparison to those who have never been exposed to antiresorptives. Continuous antiresorptive therapy reduces bone turnover and fracture odds, whereas these odds may return toward baseline once therapy stops. This attenuation is well documented for bisphosphonates in extension trials [11] and particularly for denosumab [27]. In addition, the gradient favoring current use may partly reflect adherence/persistence phenomena and the “healthy adherer” effect [28]: patients who persist with therapy often differ in behaviors and care patterns relevant to fracture risk, and higher adherence to antiosteoporotic medication has been associated with fewer fractures. Although covariate adjustments were applied, time-varying confounding by changes in frailty, fall risk, or concomitant medications could remain [29]. The observed exposure gradients probably reflect both biological treatment effects and residual confounding. This emphasizes the need for cautious treatment duration/adherence management [30]. However, the increased odds do not occur at cessation of treatment but are especially important in the long term after cessation of treatment (>three years).

Our observational study’s main novelty is the distinct examination of the exposures. As we mentioned before, previously published studies reported results in both directions, but the information about exposures remained unclear. In a national French cohort, hip fracture reduction with oral bisphosphonates and denosumab only appeared 18–24 months after initiation [31], in contrast to our study. However, this younger cohort included women aged ≥ 55 years. In a Medicare cohort [32], non-frailty women aged ≥ 65 years treated with osteoporosis medications for >90 days showed reduced risk of subsequent hip fracture (no reduction in frailty patients). Our finding regarding the effect of a continuous/accumulated duration of more than three years in increasing hip fracture risk is inconsistent with current evidence based on clinical trials. These trials suggest that postmenopausal women with osteoporosis would need to be treated with a bisphosphonate for 20.3 months (95% CI, 11.0–29.7 months) to prevent 1 hip fracture [13]. This long period of time to find effectiveness would be related to the slower effect of the antiresorptive on cortical bone (which is fundamental in the strength of the femoral neck) [25]. Moreover, when the adherence to antiresorptive medications increases, effectiveness of the medication would increase in the same way [33,34].

When analyzing the odds of hip fracture according to the interval between the diagnosis of osteoporosis and hip fracture, it was observed that only in the groups with less than two years (imminent risk of fracture [35]) and two to five years [17] was there an increased odds of fracture if the patient was not currently exposed to an antiresorptive. These findings contrast with other cohorts where benefits from treatment for more than five years have been observed [36]. These differences are related to the variability in differences in study populations, baseline fracture risk, and variations in adherence to treatment protocols [20]. Additionally, the effectiveness of antiresorptive treatment may diminish in delay initiation [37] due to the changes in basal risk, highlighting the importance of personalized medicine in managing this condition.

Secondary prevention is the scenario where antiresorptive therapies have traditionally demonstrated the most substantial and clear efficacy in preventing hip fractures. Our cohort reflects these findings. This is related to the fact that this group is at very high risk with a wide margin for improvement with the appropriate treatment [38] with easily identification by guidelines and healthcare providers [4,5] and better motivation for adherence [39]. However, in our cohort, we could not find similar results with the main analysis due to the lack of a population in our cohort. Nevertheless, current users had lower odds of fractures compared to never users according to the moment of exposure to antiresorptives.

Related to very older adults, few cohort studies have shown positive effects of antiresorptives on the risk of hip fracture in subjects at least 80 years of age [18,40], in contrast with meta-analyses of randomized controlled trials and other cohorts where this effect disappears [11,21]. These differences are probably related to the fact that many studies did not include very older adults [19,41], poor adherence in real life practice [39], and the lack of consideration of frailty in these studies [42]. In our cohort, those over 85 had similar results to the rest of the cohort, supporting similar management in this group [40]. The finding of an increased risk of fracture when any exposition of treatment was compared to never users may be related to the higher baseline fracture risk in patients who receive bisphosphonate treatment, as they are typically those with more severe osteoporosis or other risk factors for fracture [38].

Related to bisphosphonates, our study had mixed results compared with existing literature, with lower odds of hip fractures but higher odds among long-duration exposure. These findings may be related to a waning on-target effect after discontinuation, which is biologically and clinically plausible: extension trials show that antiresorptive benefits are greatest on treatment, may partially persist after stopping, and then attenuate over time [13]. Guidance on long-term bisphosphonate therapy similarly anticipates risk “drift” toward baseline with prolonged drug holidays, particularly in patients at higher underlying risk [5]. Second, adherence and persistence likely contribute: better adherence is associated with fewer fractures, whereas early discontinuation (for intolerance, polypharmacy, or perceived low benefit) may cluster with higher-risk profiles [43]. Although we applied multivariable adjustment, residual confounding—particularly time-varying changes in frailty, falls, or corticosteroid exposure—cannot be fully excluded, and this underscores the associative (not causal) interpretation of these comparisons. Related to denosumab, our analysis demonstrated that current use of denosumab associated with lower odds of hip fractures, even more than bisphosphonates. However, this benefit rapidly diminishes after discontinuation, and the long-term deleterious effect is like bisphosphonates. Nevertheless, the rebound effect observed in long-term users after stopping denosumab treatment raises concerns about its management, suggesting a concern in long life expectancy patients [27]. Our findings reinforce current guidance to avoid unplanned denosumab interruptions and to provide a bisphosphonate “relay” when discontinuation is necessary [5]. However, these drug-specific analyses were not designed to provide causal head-to-head comparisons between antiresorptive classes, because treatment selection is influenced by patient characteristics that are incompletely captured in routinely collected data.

Related to switchers, it is known as a complex matter [44] with mixed results of maintaining BMD gains and fracture prevention. Careful attention to osteoporosis medication transitions can mitigate bone density loss and its consequences [45]. The complexities and lack of consensus related to this scenario may affect the results in our cohort, with no clear benefit of treatment in this group. New sequential guidelines are needed to be followed in order to better fracture prevention [38], and most probably lifestyle interventions should be prioritized.

### Strengths and Limitations

This research possesses multiple strengths. A significant advantage is the incorporation of a broad and varied group of older women with extensive variables and exposures. The utilization of centralized electronic health records allowed for precise measurement of antiresorptive usage, occurrences of hip fractures, and an extensive range of potential confounders that vary over time. These real-world data reflect actual clinical practices, which can sometimes differ from the controlled conditions of randomized clinical trials, and strengthen the relevance of the findings for healthcare professionals and policymakers aiming to improve osteoporosis management strategies. Statistical techniques were employed to adjust for a wide array of possible variables that might influence both the likelihood of continuing treatment and the risk of hip fractures. The stratification of the analysis by treatment duration (continuous and cumulative use) offers valuable insights into the long-term effects of antiresorptive therapies. This approach helps address important clinical questions about the optimal duration of therapy and the risks associated with prolonged use or discontinuation of treatment, particularly in older and frail populations.

One of the major limitations in this type of cohort and in the management of osteoporosis is the inherent limitations of observational research using routinely collected data. First, selection bias cannot be fully excluded despite incidence-density sampling and matching, and residual imbalances may persist after multivariable adjustment [29]. Second, exposure and covariate misclassification are possible because primary care records may be incomplete or imprecise. Although such misclassification is plausibly non-differential with respect to hip fracture and would tend to bias associations toward the null, differential errors cannot be ruled out. In our study, cases were marginally older, had more comorbidity, and more often used fall-risk-increasing drugs—including benzodiazepines, antidepressants, antipsychotics, antiepileptics, and systemic corticosteroids—which are established predictors of falls and fractures in older adults [46]. Although our models adjusted for these factors and additional frailty proxies, residual confounding by unmeasured severity or functional status may remain. Another limitation is the lack of consideration for conditions of great importance both for the initiation of treatment and for the occurrence of new fractures, such as frailty [42]. Frailty status is likely to be a useful prognostic variable in guiding patient management and in stratifying clinical benchmarks and research trials [47]. Moreover, this group of patients experienced poorer outcomes and were excluded from the dedicated care pathways that hip fracture patients received [48]. As we can observe in previous cohorts, control groups usually have older, impaired, and unhealthy patients [18], making the validity of these findings questionable. It is known that prescribing antiresorptives to individuals who are expected to have a long life expectancy and health status has an impact in cohort studies [49]. Despite adjustments made in matching and analysis for potential confounding factors, it is important to analyze these data in cohorts that take frailty into consideration. Nevertheless, the possibility of unidentified biases remains a significant concern, and the possibility of residual cofounding cannot be completely ruled out. Crucially, the increased risk of hip fracture observed in long-term continuous users and past users (>three years) must be interpreted with extreme caution due to the high likelihood of confounding by indication and severity. Patients who are prescribed antiresorptive therapy for extended periods typically possess a more severe baseline osteoporosis profile compared to ‘never users’ [4]. While our models adjusted for measurable confounders, this analytical conditioning cannot fully remove the net bias if unmeasured variables, such as inherent disease severity, remain [50]. Therefore, the higher fracture risk in these groups should be viewed primarily as a marker of underlying disease severity rather than a direct deleterious effect of the medication. Additionally, as BIFAP relies on clinical diagnostic codes recorded in primary care, specific quantitative values from DXA, such as exact BMD T-scores, are not systematically available for extraction. Consequently, we could not adjust our models for absolute baseline bone mass [23,36]. We also cannot entirely rule out survival bias, wherein individuals prescribed long-term therapies may survive long enough to experience an age-related fracture that would have otherwise been preempted by competing mortality risks [42,49]. Finally, as this population-based study utilizes data exclusively from the Spanish healthcare system, our findings may not be fully generalizable to other populations with different genetic backgrounds, baseline fracture risks, or healthcare delivery models [51].

## 5. Conclusions

In this population-based nested case–control study of women aged ≥ 75 years with osteoporosis, current antiresorptive use was associated with lower odds of incident hip fracture, whereas past use was associated with higher odds compared with never use. No overall association was observed when ever users were compared with never users. These findings are compatible with time-dependent patterns of treatment exposure, persistence, and discontinuation in routine clinical practice, but they should not be interpreted as causal evidence of benefit or harm because of potential confounding by indication, disease severity, frailty, and differential clinical monitoring. Clinically, the results support careful follow-up of very old women receiving or discontinuing antiresorptive therapy, avoidance of unplanned treatment interruptions—particularly for denosumab—and individualized decision-making based on fracture risk, comorbidity, frailty, tolerability, and patient preferences.

## Figures and Tables

**Table 1:** Baseline characteristics of cases and controls.

Variables	Cases	Controls	*p*-Value
N	2057	19,275	
Age (years), mean (SD)	87.5 (5.8)	87.2 (5.1)	0.007 ^1^
Follow-up from cohort entry to index date (months), mean (SD)	86.6 (33.6)	52.8 (27.2)	<0.001 ^1^
**Comorbidities, n (%)**			
	1537 (74.7%)	13,631 (70.7%)	<0.001 ^2^
Hypertension	1054 (51.2%)	9992 (51.8%)	0.605 ^2^
Ischemic heart disease	35 (1.7%)	305 (1.6%)	0.682 ^2^
Alcohol abuse	5 (0.2%)	42 (0.2%)	0.817 ^2^
Anxiety	68 (3.3%)	661 (3.4%)	0.769 ^2^
Atrial fibrillation	465 (22.6%)	3805 (19.7%)	0.002 ^2^
Asthma	68 (3.3%)	661 (3.4%)	0.769 ^2^
Celiac disease	6 (0.3%)	18 (0.1%)	0.011 ^2^
Diabetes	325 (15.8%)	2974 (15.4%)	0.659 ^2^
Dementia	319 (15.5%)	2344 (12.2%)	<0.001 ^2^
Depression	366 (17.8%)	2511 (13.0%)	<0.001 ^2^
Parkinson’s disease	27 (1.3%)	242 (1.3%)	0.825 ^2^
Stroke	260 (12.6%)	2039 (10.6%)	0.004 ^2^
Hypothyroidism	198 (9.6%)	1865 (9.7%)	0.942 ^2^
Heart failure	412 (20.0%)	4181 (21.7%)	0.081 ^2^
Renal impartment	291 (14.1%)	2824 (14.7%)	0.538 ^2^
Tobacco	16 (0.8%)	56 (0.3%)	<0.001 ^2^
COPD	3 (0.1%)	30 (0.2%)	1.0 ^3^
Drug abuse	0 (0.0%)	2 (0.0%)	1.0 ^3^
**Current use of, n (%)**			
Anxiolytics derivates benzodiazepines	1320 (64.2%)	9875 (51.2%)	<0.001 ^2^
Anxiolytics others	71 (3.5%)	466 (2.4%)	0.004 ^2^
Antagonists H2	111 (5.4%)	773 (4.0%)	0.003 ^2^
Antiepileptics	371 (18.0%)	3081 (16.0%)	0.016 ^2^
Antidepressants	1057 (51.4%)	8023 (41.6%)	<0.001 ^2^
Antihypertensives	70 (3.4%)	514 (2.7%)	0.052 ^2^
Antipsychotics	513 (24.9%)	3986 (20.7%)	<0.001 ^2^
Bazedoxifene	13 (0.6%)	46 (0.2%)	0.001 ^2^
Calcium	86 (4.2%)	712 (3.7%)	0.269 ^2^
Calcitonin	33 (1.6%)	134 (0.7%)	<0.001 ^2^
Systemic corticoids	432 (21.0%)	3004 (15.6%)	<0.001 ^2^
Loop diuretics	891 (43.3%)	8112 (42.1%)	0.283 ^2^
Statins	909 (44.2%)	7299 (37.9%)	<0.001 ^2^
Proton pump inhibitors	1663 (80.8%)	13,898 (72.1%)	<0.001 ^2^
Lithium	2 (0.1%)	9 (0.05%)	0.337 ^2^
Insulin	97 (4.7%)	1022 (5.3%)	0.257 ^2^
Glucose-lowering drugs	381 (18.5%)	3346 (17.4%)	0.187 ^2^
Raloxifene	12 (0.6%)	81 (0.4%)	0.286 ^2^
Ranelate of strontium	62 (3.0%)	322 (1.7%)	<0.001 ^2^
Vitamin D	564 (27.4%)	5878 (30.5%)	0.004 ^2^
Hormonal substitutive treatment	0 (0.0%)	0 (0.0%)	
Teriparatide	80 (3.9%)	560 (2.9%)	0.013 ^2^
Thiazolidinediones	4 (0.2%)	29 (0.2%)	0.629 ^2^
**Fracture prevalence, n (%)**			
Humerus	120 (5.8%)	857 (4.4%)	0.004 ^2^
Vertebral	131 (6.4%)	1318 (6.8%)	0.421 ^2^
Hand	21 (1.0%)	184 (1.0%)	0.770 ^2^
Others	129 (6.3%)	1216 (6.3%)	0.947 ^2^
Radius	79 (3.8%)	618 (3.2%)	0.124 ^2^
Tibius	58 (2.8%)	417 (2.2%)	0.055 ^2^
Foot	30 (1.5%)	262 (1.4%)	0.713 ^2^
**Drug exposure from inclusion to the index date, n (%)**			
	667 (32.4%)	5514 (28.6%)	<0.001 ^2^
Bisphosphonates	536 (26.1%)	3621 (18.8%)	<0.001 ^2^
Alendronate	292 (14.2%)	2259 (11.7%)	0.001 ^2^
Clodronate	0 (0.0%)	0 (0.0%)	
Denosumab	159 (7.7%)	2023 (10.5%)	<0.001 ^2^
Etidronate	1 (0.0%)	2 (0.0%)	0.262 ^3^
Ibandronate	136 (6.6%)	654 (3.4%)	<0.001 ^2^
Pamidronate	0 (0.0%)	0 (0.0%)	
Risedronate	139 (6.8%)	814 (4.2%)	<0.001 ^2^
Tiludronate	0 (0.0%)	0 (0.0%)	
Zoledronic	1 (0.0%)	0 (0.0%)	0.096 ^2^

^1^ *t*-test; ^2^ Chi-square test; ^3^ Fisher’s exact test.

**Table 2:** Association between exposure to antiresorptives and the risk of hip fracture.

	Cases (n = 2057, 9.65%)	Controls (n = 19,257, 90.35%)	OR (95% CI)	Adjusted OR (95% CI)
Current use versus past or never use ^1^				
Current users	301 (6.9%)	4039 (93.1%)	ref	ref
Past users + never users	1606 (10%)	14,379 (90%)	1.26 (1.09, 1.44)	1.43 (1.24, 1.65)
Current use versus non-current or never use				
Current users	301 (6.9%)	4039 (93.1%)	ref	ref
Past users + recent users + never users	1756 (10.3%)	15,236 (89.7%)	1.27 (1.11, 1.46)	1.35 (1.17, 1.55)
Ever use versus never use ^2^				
Never users	1390 (9.2%)	13,761 (90.8%)	ref	ref
Ever users	667 (10.8%)	5514 (89.2%)	1.14 (1.02, 1.27)	1.01 (0.90, 1.13)
Timing of antiresorptive exposure (risk associated with treatment discontinuation)				
Current users	301 (6.9%)	4039 (93.1%)	ref	ref
Recent users	150 (14.9%)	857 (85.1%)	1.51 (1.21, 1.89)	1.48 (1.18, 1.86)
Past users	216 (25.9%)	618 (74.1%)	2.40 (1.93, 2.98)	2.54 (2.03, 3.16)
Never users	1390 (9.2%)	13,761 (90.8%)	1.17 (1.01, 1.33)	1.33 (1.15, 1.53)
According to the moment of exposure to antiresorptive (risk associated with treatment exposure)				
Never user	1390 (9.2%)	13,761 (90.8%)	ref	ref
Current user	301 (6.9%)	4039 (93.1%)	0.86 (0.75, 0.99)	0.76 (0.65, 0.87)
Recent users	150 (14.9%)	857 (85.1%)	1.30 (1.07, 1.57)	1.12 (0.91, 1.37)
Past users	216 (25.9%)	618 (74.1%)	2.06 (1.70, 2.48)	1.91 (1.57, 2.33)
According to the duration of exposure to antiresorptive				
Continuous duration of antiresorptive				
Never user	1390 (9.2%)	13,761 (90.8%)	ref	ref
≤1 year	297 (9.1%)	2955 (90.9%)	1.07 (0.92, 1.23)	1.02 (0.88, 1.18)
>1 and <3 years	254 (10.9%)	2086 (89.1%)	1.09 (0.93, 1.27)	1.03 (0.88, 1.21)
>3 years	116 (19.7%)	473 (80.3%)	1.66 (1.32, 2.10)	1.48 (1.17, 1.88)
Cumulative duration of antiresorptive				
Never user	1390 (9.2%)	13,761 (90.8%)	ref	ref
≤1 year	325 (8.6%)	3463 (91.4%)	1.01 (0.88, 1.16)	0.97 (0.84, 1.16)
>1 and <3 years	221 (11.6%)	1678 (88.4%)	1.10 (0.93, 1.29)	1.03 (0.87, 1.22)
>3 years	121 (24.5%)	373 (75.5%)	2.17 (1.71, 2.76)	1.97 (1.55, 2.52)
According to the interval between OP diagnosis and hip fracture				
<2 years n = 11,397				
Current users	66 (2.5%)	2594 (97.5%)	ref	ref
PU + RU + NU	328 (3.8%)	8409 (96.2%)	1.44 (1.07, 1.93)	1.48 (1.10, 2.00)
2–5 years n = 7076				
Current users	126 (9.8%)	1154 (90.2%)	ref	ref
PU + RU + NU	713 (12.3%)	5083 (87.7%)	1.22 (0.98, 1.52)	1.34 (1.06, 1.69)
>5 years n = 2859				
Current users	109 (27.3%)	291 (72.8%)	ref	ref
PU + RU + NU	715 (29.1%)	1744 (70.9%)	1.14 (0.86, 1.50)	1.13 (0.84, 1.51)

^1^ Recent users were excluded from this comparison. ^2^ Ever users included current, recent, and past users.

**Table 3:** Association between exposure to antiresorptives and the risk of hip fracture in patients with previous fractures (secondary prevention).

	Cases (n = 525, 9.5%)	Controls (n = 5005, 90.5%)	OR (95% CI)	Adjusted OR (95% CI)
Current use versus past or never use ^1^				
Current users	85 (6.4%)	1251 (93.6%)	ref	ref
Past users + never users	397 (10.1%)	3534 (89.9%)	1.53 (1.14, 2.06)	1.73 (1.25, 2.40)
Current use versus non-current or never use				
Current users	85 (6.4%)	1251 (93.6%)	ref	ref
Past users + recent users + never users	440 (10.5%)	3754 (89.5%)	1.55 (1.16, 2.06)	1.66 (1.22, 2.25)
Ever use versus never use ^2^				
Never users	349 (9.2%)	3425 (90.8%)	ref	ref
Ever users	176 (10.0%)	1580 (90.0%)	0.99 (0.78, 1.25)	0.88 (0.68, 1.15)
Timing of antiresorptive exposure (risk associated with treatment discontinuation)				
Current users	85 (6.4%)	1251 (93.6%)	ref	ref
Recent users	43 (16.3%)	220 (83.7%)	1.85 (1.17, 2.92)	1.59 (0.98, 2.58)
Past users	48 (30.6%)	109 (69.4%)	3.28 (1.94, 5.56)	3.76 (2.18, 6.50)
Never users	349 (9.2%)	3425 (90.8%)	1.43 (1.07, 1.92)	1.58 (1.15, 2.18)
According to the moment of exposure to antiresorptive (risk associated with treatment exposure)				
Never users	349 (9.2%)	3425 (90.8%)	ref	ref
Current user	85 (6.4%)	1251 (93.6%)	0.70 (0.52, 0.84)	0.63 (0.46, 0.87)
Recent users	43 (16.3%)	220 (83.7%)	1.29 (0.86, 1.93)	1.00 (0.65, 1.56)
Past users	48 (30.6%)	109 (69.4%)	2.29 (1.43, 3.68)	2.38 (1.44, 3.91)
According to the duration of exposure to antiresorptive				
Continuous duration of antiresorptive				
Never users	349 (9.2%)	3425 (90.8%)	ref	ref
≤1 year	81 (8.7%)	850 (91.3%)	0.95 (0.70, 1.28)	0.89 (0.65, 1.23)
>1 and <3 years	65 (9.6%)	609 (90.4%)	0.92 (0.66, 1.28)	0.90 (0.63, 1.28)
>3 years	30 (19.9%)	121 (80.1%)	1.46 (0.86, 2.47)	1.29 (0.73, 2.29)
Cumulative duration of antiresorptive				
Never users	349 (9.2%)	3425 (90.8%)	ref	ref
≤1 year	87 (8.0%)	1005 (92.0%)	0.90 (0.67, 1.20)	0.84 (0.62, 1.15)
>1 and <3 years	65 (12.2%)	467 (87.8%)	1.05 (0.75, 1.48)	1.03 (0.71, 1.48)
>3 years	24 (18.2%)	108 (81.8%)	1.39 (0.78, 2.49)	1.27 (0.68, 2.38)
According to the interval between OP diagnosis and hip fracture				
<2 years n = 3283				
Current users	27 (3.1%)	854 (96.9%)	ref	ref
PU + RU + NU	90 (3.7%)	2312 (96.3%)	1.24 (0.68, 2.24)	1.27 (0.63, 2.59)
2–5 years n = 1705				
Current users	41 (11.0%)	333 (89.0%)	ref	ref
PU + RU + NU	202 (15.2%)	1129 (84.8%)	1.51 (0.95, 2.38)	1.47 (0.88, 2.45)
>5 years n = 542				
Current users	17 (21.0%)	64 (79.0%)	ref	ref
PU + RU + NU	148 (32.1%)	313 (67.9%)	1.97 (0.68, 5.73)	2.61 (0.57, 11.9)

^1^ Recent users were excluded from this comparison. ^2^ Ever users included current, recent, and past users.

## Data Availability

All the BIFAP Program data are broadly available via the BIFAP Program Researcher Workbench to registered users.

## Supplementary Materials

The following supporting information can be downloaded at: https://www.mdpi.com/article/10.3390/jcm15166224/s1. Table S1. Internal Classification of Diseases (ICD) codes for osteoporosis (OP); Table S2. Internal Classification of Diseases (ICD) codes for hip fracture; Table S3. Anatomical, Therapeutic, Chemical classification system (ATC) codes for antiresorptive; Table S4. Anatomical, Therapeutic, Chemical classification system (ATC) codes for adjustment drugs; Table S5. Association between exposure to antiresorptive and the risk of hip fractures in patients ≥85 years; Table S6. Association between exposure to bisphosphonates and the risk of hip fracture; Table S7. Association between exposure to denosumab and the risk of hip fracture; Table S8. Association between exposure to antiresorptive and the risk of hip fracture in switcher patients (defined as taking two or more antiresorptives during the follow-up); Figure S1. Illustration of different exposure definitions in the cohort.

## Abbreviations

The following abbreviations are used in this manuscript:

AEMPS	Spanish Agency of Medicines and Medical Devices
aOR	Adjusted odds ratio
ATC	Anatomical Therapeutic Chemical classification system
BIFAP	Base de Datos para la Investigación Farmacoepidemiológica en Ámbito Público
BMD	Bone mineral density
CI	Confidence interval
CIBERFES	CIBER of Frailty and Healthy Aging
CMBD	Conjunto Mínimo Básico de Datos (Register of Specialised Care Activity)
COPD	Chronic obstructive pulmonary disease
CU	Current user
DXA	Dual-energy X-ray absorptiometry
ENCePP	European Network of Centres for Pharmacoepidemiology and Pharmacovigilance
HUN	Hospital Universitario de Navarra
NU	Never user
OP	Osteoporosis
OR	Odds ratio
PU	Past user
RU	Recent user
SD	Standard deviation
STROBE	Strengthening the Reporting of Observational Studies in Epidemiology
