# Supplementary material for: Antiresorptive Use and Risk of Hip Fracture in Older Women with Osteoporosis: Findings from a Real-World National Database of 21,332 Women
Source: J Clin Med. 2026 Aug 11;15(16):6224. doi: 10.3390/jcm15166224 (PMC13513183; doi:10.3390/jcm15166224)
Supplement: Supplementary file 1 [file jcm-15-06224-s001.zip › jcm-4428852-supplementary.pdf]

Table S1. Internal Classification of Diseases (ICD) codes for osteoporosis (OP)

Classification	Code	Description
**ICD-9**	733.0	Osteoporosis
**ICD-10**	M80.0	Age-related osteoporosis with current pathological fracture
	M81.0	Age-related osteoporosis without current pathological fracture

Table S2. Internal Classification of Diseases (ICD) codes for hip fracture

Classification	Code	Description
**ICD-9**	820	Fracture of neck of femur
	821	Fracture of other and unspecified parts of femur
	V54.13	Aftercare for healing traumatic fracture of hip
	V54.15	Aftercare for healing traumatic fracture of upper leg
**ICD-10**	S72	Fracture of femur
	S72.0	Fracture of head and neck of femur
	S72.1	Pertrochanteric fracture
	S72.2	Subtrochanteric fracture of femur

Table S3. Anatomical, Therapeutic, Chemical classification system (ATC) codes for antiresorptive.

Antiresorptive	ATC	ATC combinations
Bisphosphonates	M05BA	
Alendronate	M05BA04	M05BB03, M05BB05, M05BB06
Clodronate	M05BA02	
Denosumab	M05BX04	
Etidronate	M05BA01	M05BB01
Ibandronate	M05BA06	M05BB09
Pamidronate	M05BA03	
Risedronate	M05BA07	M05BB02, M05BB04, M05BB07
Tiludronate	M05BA05	
Zoledronic	M05BA08	M05BB08

Table S4. Anatomical, Therapeutic, Chemical classification system (ATC) codes for adjustment drugs.

Drugs	ATC
Anxiolytics derivates benzodiazepines	N05BA
Anxiolytics others	N05BB, N05BC, N05BD, N05BE, N05BX
Antagonists H2	A02BA
Antiepileptics	N03A
Antidepressive	N06A
Antihypertensives	M05BA

Antipsychotics	N05A
Bazedoxifene	G03XC02
Calcium	A12AA, A12AX
Calcitonin	H05BA
Corticoids systemics	H02A, H02B
Loop diuretics	C03C
Statins	C10AA, C10BA
Inhibitors of bomb protons	A02BC
Lithium	N05AN01
Hipoglucemic insulin	A10A
Hipoglucemics no insulin	A10B
Raloxifene	G03XC01
Ranelate of strontium	M05BX53
Supplements alimentations	V06
Proteins Supplements	V06B
Vitamin D	A11CC
Hormonal substitutive treatment	G03AA
Teriparatide	H05AA02
Thiazolidinediones	A10BG

Table S5. Association between exposure to antiresorptive and the risk of hip fractures in patients ≥85 years

	Cases (n=1474, 10.2%)	Controls (n=13045, 89.8%)	OR (95% CI)	Adjusted OR (95% CI)
Current use versus past or never use[1]				
Current users	176 (7.0%)	2345 (93.0%)	ref	ref
past users + never users	1204 (10.59%)	10162 (89.41%)	1.36 (1.13, 1.62)	1.42 (1.17, 1.72)
Current use versus non-current or never use				
Current users	176 (7.0%)	2345 (93.0%)	ref	ref
past users + recent users + never users	1298 (10.8%)	10700 (89.2%)	1.34 (1.12, 1.59)	1.40 (1.16, 1.69)
Ever use versus never use[2]				
Never users	1030 (9.6%)	9701 (90.4%)	ref	ref
Ever users	444 (11.7%)	3344 (88.3%)	1.16 (1.01, 1.32)	1.45 (1.10, 1.90)
Timing of antiresorptive exposure				
Current users	176 (7.0%)	2345 (93.0%)	ref	ref
Recent users	94 (14.9%)	538 (85.1%)	1.45 (1.09, 1.92)	1.29 (0.96, 1.75)
Past users	174 (27.4%)	461 (72.6%)	2.73 (2.10, 3.54)	2.18 (1.64, 2.89)
Never users	1030 (9.6%)	9701 (90.4%)	1.22 (1.02, 1.46)	1.32 (1.09, 1.61)
According to the moment of exposure to antiresoptive				
Never users	1030 (9.6%)	9701 (90.4%)	ref	ref

Current users	176 (7.0%)	2345 (93.0%)	0.82 (0.68, 0.98)	0.76 (0.62, 0.92)
Recent users	94 (14.9%)	538 (85.1%)	1.19 (0.93, 1.51)	0.98 (0.75, 1.27)
Past users	174 (27.4%)	461 (72.6%)	2.23 (1.80, 2.77)	1.65 (1.29, 2.10)
According to the duration of exposure to antiresorptive				
Continuous duration of Antiresorptive				
Never users	1030 (9.6%)	9701 (90.4%)	ref	ref
≤1 year	199 (10.0%)	1796 (90.0%)	1.10 (0.93, 1.32)	1.04 (0.87, 1.25)
>1 and < 3 years	164 (11.8%)	1227 (88.2%)	1.06 (0.88, 1.28)	1.00 (0.82, 1.22)
>3 years	81 (20.1%)	321 (79.9%)	1.68 (1.27, 2.22)	1.51 (1.14, 2.01)
Cumulative duration of Antiresorptive				
Never users	1030 (9.6%)	9701 (90.4%)	ref	ref
≤1 year	209 (9.2%)	2062 (90.8%)	1.03 (0.87, 1.22)	0.98 (0.82, 1.17)
>1 and < 3 years	145 (12.5%)	1012 (87.5%)	1.07 (0.88, 1.31)	1.00 (0.81, 1.23)
>3 years	90 (25.0%)	270 (75.0%)	2.16 (1.63, 2.85)	2.01 (1.51, 2.67)
According interval between OP diagnosis and hip fracture				
<2 years n=7287				
Current users	31 (2.1%)	1452 (97.9%)	ref	ref
PU+RU+NU	217 (3.7%)	5587 (96.3%)	1.49 (0.98, 2.25)	1.42 (0.92, 2.22)
2-5 years n=4848				
Current users	64 (8.6%)	683 (91.4%)	ref	ref
PU+RU+NU	484 (11.8%)	3617 (88.2%)	1.39 (1.03, 1.87)	1.35 (0.98, 1.85)
>5 years n=2384				
Current users	81 (27.8%)	210 (72.2%)	ref	ref
PU+RU+NU	597 (28.5%)	1496 (71.5%)	1.09 (0.79, 1.50)	1.09 (0.77, 1.54)

1 Recent users were excluded from this comparison.

2 Ever users included current, recent, and past users.

Table S6. Association between exposure to bisphosphonates and the risk of hip fracture

	Cases (n=1945, 9.45%)	Controls (n=18620, 90.55%)	OR (95% CI)	Adjusted OR (95% CI)
Current use versus past or never use[1]				
Current users	220 (8.4%)	2399 (91.6%)	ref	ref
past users + never users	1725 (9.61%)	16221 (90.39%)	1.15 (0.98, 1.35)	1.19 (1.01, 1.40)
Current use versus non-current or never use				
Current users	220 (8.4%)	2399 (91.6%)	ref	ref
past users + recent users + never users	1837 (9.8%)	16876 (90.2%)	1.14 (0.98, 1.34)	1.18 (1.01, 1.39)
Ever use versus never use[2]				
Never users	1521 (8.9%)	15654 (91.1%)	ref	ref
Ever users	536 (12.9%)	3621 (87.1%)	1.24 (1.10, 1.40)	1.20 (1.06, 1.35)
Timing of antiresorptive exposure				
Current users	220 (8.4%)	2399 (91.6%)	ref	ref
Recent users	112 (14.6%)	655 (85.4%)	1.26 (0.97, 1.63)	1.08 (0.92, 1.28)
Past users	204 (26.5%)	567 (73.5%)	2.24 (1.77, 2.82)	1.24 (0.96, 1.62)
Never users	1521 (8.9%)	15654 (91.1%)	1.05 (0.89, 1.23)	2.18 (1.72, 2.76)

According to the moment of exposure to antiresoptive				
Never user	1521 (8.9%)	15654 (91.1%)	ref	ref
Current user	220 (8.4%)	2399 (91.6%)	0.95 (0.81, 1.12)	0.92 (0.78, 1.09)
Recent users	112 (14.6%)	655 (85.4%)	1.20 (0.96, 1.50)	1.15 (0.92, 1.44)
Past users	204 (26.5%)	567 (73.5%)	2.13 (1.75, 2.59)	2.01 (1.65, 2.45)
According to the duration of exposure to antiresorptive				
Continuous duration of Antiresorptive				
Never user	1521 (8.9%)	15654 (91.1%)	ref	ref
≤1 year	260 (11.4%)	2030 (88.6%)	1.18 (1.01, 1.38)	1.14 (0.87, 1.33)
>1 and < 3 years	185 (12.7%)	1269 (87.3%)	1.17 (0.98, 1.40)	1.16 (0.96, 1.39)
>3 years	90 (21.8%)	322 (78.2%)	1.74 (1.34, 2.27)	1.58 (1.20, 2.07)
Cumulative duration of Antiresorptive				
Never user	1521 (8.9%)	15654 (91.1%)	ref	ref
≤1 year	277 (10.4%)	2382 (89.6%)	1.10 (0.95, 1.27)	1.06 (0.92, 1.24)
>1 and < 3 years	166 (14.0%)	1018 (86.0%)	1.21 (1.00, 1.46)	1.17 (0.96, 1.42)
>3 years	92 (29.4%)	221 (70.6%)	2.54 (1.92, 3.36)	2.35 (1.77, 3.14)
According interval between OP diagnosis and hip fracture				
<2 years				
Current users	33 (2.2%)	1441 (97.8%)	ref	ref
PU+RU+NU	361 (3.6%)	9562 (96.4%)	1.58 (1.08, 2.34)	1.64 (1.10, 2.43)
2-5 years				
Current users	88 (10.5%)	748 (89.5%)	ref	ref
PU+RU+NU	751 (12.0%)	5489 (88.0%)	1.14 (0.88, 1.48)	1.24 (0.95, 1.63)
>5 years				
Current users	99 (32.0%)	210 (68.0%)	ref	ref
PU+RU+NU	725 (28.4%)	1825 (71.6%)	0.88 (0.65, 1.19)	0.87 (0.63, 1.19)

1 Recent users were excluded from this comparison.

2 Ever users included current, recent, and past users.

Table S7. Association between exposure to denosumab and the risk of hip fracture

	Cases (n=2018, 9.57%)	Controls (n=19060, 90.43%)	OR (95% CI)	Adjusted OR (95% CI)
Current use versus past or never use[1]				
Current users	99 (5.4%)	1740 (94.6%)	ref	ref
past users + never users	1919 (9.97%)	17320 (90.03%)	1.38 (1.10, 1.72)	1.50 (1.20, 1.89)
Current use versus non-current or never use				
Current users	99 (5.4%)	1740 (94.6%)	ref	ref
past users + recent users + never users	1958 (10.0%)	17535 (90.0%)	1.39 (1.11, 1.74)	1.52 (1.21, 1.91)
Ever use versus never use[2]				
Never users	1898 (9.9%)	17252 (90.1%)	ref	ref
Ever users	159 (7.3%)	2023 (92.7%)	0.91 (0.76, 1.10)	0.83 (0.68, 0.99)
Timing of antiresorptive exposure				
Current users	99 (5.4%)	1740 (94.6%)	ref	ref
Recent users	39 (15.4%)	215 (84.6%)	2.22 (1.45, 3.40)	2.16 (1.41, 3.33)
Past users	21 (23.6%)	68 (76.4%)	2.17 (1.20, 3.92)	2.00 (1.10, 3.64)
Never users	1898 (9.9%)	17252 (90.1%)	1.37 (1.10, 1.71)	1.50 (1.19, 1.88)

According to the moment of exposure to antiresoptive				
Never users	1898 (9.9%)	17252 (90.1%)	ref	ref
Current user	99 (5.4%)	1740 (94.6%)	0.73 (0.58, 0.91)	0.67 (0.53, 0.84)
Recent users	39 (15.4%)	215 (84.6%)	1.62 (1.11, 2.35)	1.45 (0.99, 2.11)
Past users	21 (23.6%)	68 (76.4%)	1.59 (0.91, 2.75)	1.34 (0.76, 2.34)
According to the duration of exposure to antiresorptive				
Continuous duration of Antiresorptive				
Never users	1898 (9.9%)	17252 (90.1%)	ref	ref
≤1 year	62 (5.6%)	1048 (94.4%)	0.82 (0.62, 1.08)	0.75 (0.56, 0.99)
>1 and < 3 years	76 (8.3%)	835 (91.7%)	0.92 (0.71, 1.19)	0.83 (0.63, 1.08)
>3 years	21 (13.0%)	140 (87.0%)	1.38 (0.83, 2.30)	1.21 (0.72, 2.02)
Cumulative duration of Antiresorptive				
Never users	1898 (9.9%)	17252 (90.1%)	ref	ref
≤1 year	75 (5.8%)	1221 (94.2%)	0.83 (0.65, 1.07)	0.77 (0.59, 0.99)
>1 and < 3 years	57 (7.9%)	662 (92.1%)	0.87 (0.64, 1.17)	0.77 (0.57, 1.04)
>3 years	27 (16.2%)	140 (83.8%)	1.53 (0.96, 2.42)	1.38 (0.87, 2.19)
According interval between OP diagnosis and hip fracture				
<2 years				
Current users	36 (3.0%)	1177 (97.0%)	ref	ref
PU+RU+NU	358 (3.5%)	9826 (96.5%)	1.11 (0.75, 1.64)	1.08 (0.73, 1.60)
2-5 years				
Current users	43 (8.6%)	456 (91.4%)	ref	ref
PU+RU+NU	796 (12.1%)	5781 (87.9%)	1.35 (0.94, 1.92)	1.48 (1.03, 2.14)
>5 years				
Current users	20 (15.7%)	107 (84.3%)	ref	ref
PU+RU+NU	804 (29.4%)	1928 (70.6%)	2.15 (1.24, 3.75)	2.33 (1.30, 4.18)

1 Recent users were excluded from this comparison.

2 Ever users included current, recent, and past users.

Table S8. Association between exposure to antiresorptive and the risk of hip fracture in switcher patients (defined as taking two or more antiresorptives during the follow up)

	Cases (n=1437, 9.37%)	Controls (n=13954, 90.63%)	OR (95% CI)	Adjusted OR (95% CI)
Current use versus past or never use[1]				
Current users	29 (15.8%)	156 (84.2%)	ref	ref
past users + never users	1408 (9.26%)	13798 (90.74%)	0.81 (0.52, 1.28)	0.94 (0.59, 1.50)
Current use versus non-current or never use				
Current users	29 (15.8%)	156 (84.2%)	ref	ref
past users + recent users + never users	1417 (9.3%)	13832 (90.7%)	0.82 (0.52, 1.29)	0.95 (0.60, 1.52)
Ever use versus never use[2]				
Never users	1390 (9.2%)	13761 (90.8%)	ref	ref
Ever users	56 (19.8%)	227 (80.2%)	1.64 (1.17, 2.30)	1.15 (0.81, 1.64)
Timing of antiresorptive exposure				
Current users	29 (15.8%)	156 (84.2%)	ref	ref
Recent users	9 (20.9%)	34 (79.1%)	1.69 (0.68, 4.17)	1.38 (0.53, 3.58)
Past users	18 (32.7%)	37 (67.3%)	2.15 (0.99, 4.68)	1.99 (0.90, 4.38)

Never users	1390 (9.2%)	13761 (90.8%)	0.81 (0.51, 1.27)	1.09 (0.68, 1.73)
According to the moment of exposure to antiresoptive				
Never user	1390 (9.2%)	13761 (90.8%)	ref	ref
Current user	29 (15.8%)	156 (84.2%)	1.24 (0.79, 1.96)	0.92 (0.58, 1.47)
Recent users	9 (20.9%)	34 (79.1%)	2.10 (0.95, 4.61)	1.27 (0.55, 2.95)
Past users	18 (32.7%)	37 (67.3%)	2.67 (1.41, 5.05)	1.83 (0.95, 3.52)
According to the duration of exposure to antiresorptive				
Continuous duration of Antiresorptive				
Never user	1390 (9.2%)	13761 (90.8%)	ref	ref
≤1 year	12 (16.7%)	60 (83.3%)	1.57 (0.78, 3.14)	1.42 (0.70, 2.88)
>1 and < 3 years	23 (16.8%)	114 (83.2%)	1.27 (0.77, 2.09)	1.09 (0.66, 1.82)
>3 years	21 (28.4%)	53 (71.6%)	2.57 (1.43, 4.63)	2.18 (1.19, 3.98)
Cumulative duration of Antiresorptive				
Never user	1390 (9.2%)	13761 (90.8%)	ref	ref
≤1 year	13 (14.4%)	77 (85.6%)	1.41 (0.75, 2.64)	1.31 (0.69, 2.50)
>1 and < 3 years	23 (17.0%)	112 (83.0%)	1.28 (0.78, 2.11)	1.12 (0.68, 1.85)
>3 years	20 (34.5%)	38 (65.5%)	3.35 (1.72, 6.51)	2.64 (1.33, 5.24)
According interval between OP diagnosis and hip fracture				
<2 years				
Current users	3 (6.8%)	41 (93.2%)	ref	ref
PU+RU+NU	309 (3.6%)	8186 (96.4%)	0.82 (0.17, 3.95)	0.84 (0.15, 4.67)
2-5 years				
Current users	5 (6.1%)	77 (93.9%)	ref	ref
PU+RU+NU	620 (12.4%)	4385 (87.6%)	2.12 (0.75, 5.99)	2.58 (0.89, 7.49)
>5 years				
Current users	21 (35.6%)	38 (64.4%)	ref	ref
PU+RU+NU	488 (27.9%)	1261 (72.1%)	0.59 (0.29, 1.20)	0.64 (0.30, 1.38)

1 Recent users were excluded from this comparison.

2 Ever users included current, recent, and past users.

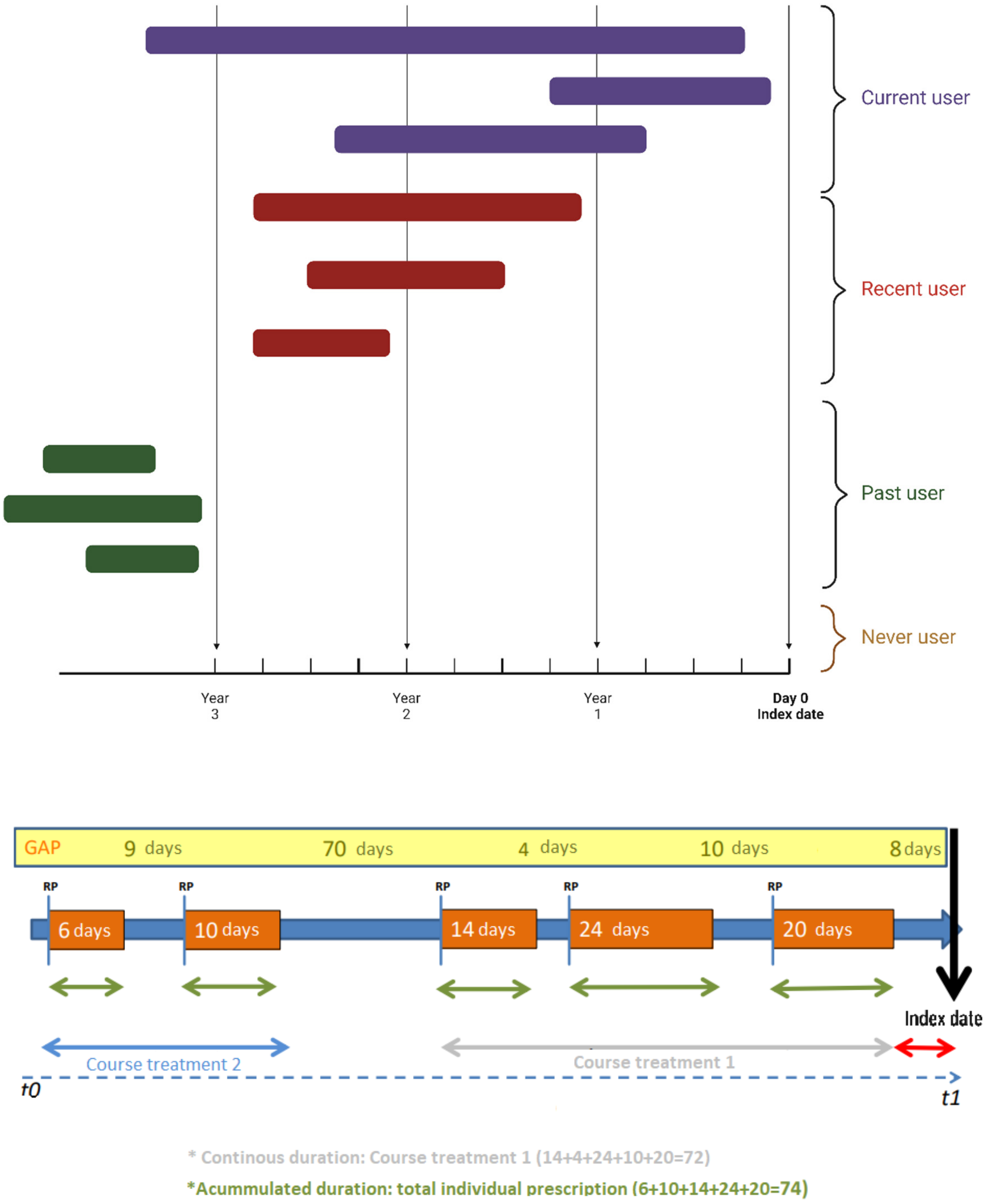

Figure S1: Illustration of different exposures definitions in the cohort.

A: Examples of different situations according to the moment of exposure to antiresorptive (both for cases and controls)

B: Example of effect of exposure time to antiresorptive. GAP: interval time between the final duration of one prescription and the beginning of a new one. RP: prescription. t0: beginning of the follow up. t1: ending of the follow up. Orange bow: time con taking the prescription
